# *Brachyspira* in dogs: risk factors of shedding in central Germany and longitudinal study of an infected kennel

**DOI:** 10.1186/s12917-024-03989-x

**Published:** 2024-04-04

**Authors:** Julia Gothe, Sarah Pfetzing, Reiner Ulrich, Wieland Schrödl, Christoph G. Baums, Romy M. Heilmann

**Affiliations:** 1 Institute of Bacteriology and Mycology, Centre for Infectious Diseases, Faculty of Veterinary Medicine, Leipzig University, Leipzig, Germany; 2 Institute of Veterinary Pathology, Faculty of Veterinary Medicine, Leipzig University, Leipzig, Germany; 3 Department for Small Animals, Veterinary Teaching Hospital, Faculty of Veterinary Medicine, University of Leipzig, An den Tierkliniken 23, 04103 Leipzig, Germany

**Keywords:** Canine intestinal spirochetosis, *Brachyspira pilosicoli*, *Brachyspira canis*, *Brachyspira pulli*, *Nox*

## Abstract

**Background:**

*Brachyspira (B.) pilosicoli* is a zoonotic pathogen, able to infect different animal species such as pigs, poultry, and rodents, causing intestinal spirochetosis. An association of gastrointestinal clinical signs, such as diarrhea, with the isolation of *B. pilosicoli* from fecal samples or rectal swabs has not been proven in dogs. Other *Brachyspira* species commonly isolated from dogs, such as “*B. canis”* and “*B. pulli”*, are considered commensals. This study investigated the occurrence of different *Brachyspira* species in rectal swabs and fecal samples in an independent canine cohort in central Germany. These included samples from shelter dogs, hunting dogs, and dogs presenting at regional small animal practices with various clinical signs. Data about the dogs, including potential risk factors for *Brachyspira* isolation, were obtained using a standardized questionnaire. The study also longitudinally investigated a colony of Beagle dogs for *Brachyspira* over 5 years.

**Results:**

The rate of *Brachyspira* spp. isolation was 11% and included different *Brachyspira* species (“*B. canis”*, “*B. pulli”*, and *B. pilosicoli*). “*B. canis*” was detected in 18 dogs, whereas *B. pilosicoli* was only isolated from 1 dog in the independent cohort (not including the Beagle colony). Risk factors for shedding *Brachyspira* and “*B. canis”* were being less than 1 year of age and shelter origin. Gastrointestinal signs were not associated with the shedding of *Brachyspira*. *B. pilosicoli* and “*B. canis*” were isolated from several dogs of the same Beagle colony in 2017 and again in 2022, while *Brachyspira* was not isolated at multiple sampling time points in 2021.

**Conclusions:**

Shedding of *B. pilosicoli* in dogs appears to be uncommon in central Germany, suggesting a low risk of zoonotic transmission from dogs. Commensal status of “*B. canis*” and “*B. pulli*” is supported by the results of this study. Findings from the longitudinal investigation of the Beagle colony agree with an asymptomatic long-term colonization of dogs with “*B. canis*” and *B. pilosicoli* and suggest that introducing new animals in a pack can trigger an increased shedding of *B. pilosicoli*.

**Supplementary Information:**

The online version contains supplementary material available at 10.1186/s12917-024-03989-x.

## Background

*Brachyspira* are anaerobic intestinal spirochetes and, due to fastidious growth in culture, can present a diagnostic challenge. *Brachyspira* show a slow, swarming growth on agar plates and need anaerobic conditions and protection from other swarming and faster-growing anaerobic bacteria. Hence, *Brachyspira* are cultivated on selective agars, typically trypticase soy agar (TSA), supplemented with 5–10% ovine or bovine blood and antibiotics to increase the likelihood of isolation and decrease the risk of overgrowth with other anaerobic fecal bacteria [1, 2]. Plates are usually incubated at 37–41 °C for 5–7 days [3, 4]. The swarming growth makes isolating different *Brachyspira* spp. from one sample challenging, often requiring multiple subcultures to attain pure cultures [5]. After microscopic detection of the spiral-shaped, Gram-negative spirochetes, differentiation of *Brachyspira* spp. can be achieved by several different methods, including matrix-assisted laser desorption ionization – time of flight mass spectrometry (MALDI-TOF MS), polymerase chain reaction (PCR), and sequencing (Table 1).

**Table 1:** Detection and differentiation of *Brachyspira (B.)* in dogs (reports published from 1989 until recently)

Country	SM^a^	Detection method						Number of samples	Positive samples, n (%)	Differentiation of Brachyspira						Reference
		TSA^b^	Mic^c^	BC^d^	PCR^e^	Seq^f^	Other			B. pilo^g^	B. canis	B. pulli	B. inno^h^	B. inter^i^	B. hyo^k^	
Germany	F^l^		x	x			CSA^m^	149	6 (4%)				5		1	[43]
Australia	F^l^	x						203	38 (19%)							[44]
					x	x	MEE^n^, ribo^o^	24/203	24		24					[21]
Papua New Guinea	R^r^	x	x		x			76	7 (9%)	4						[45]
Sweden	R^r^	x		x		x (4)	PFGE^s^ (4)	46*	24 (52%)*	2			1			[20]
Australia	F^l^	x	x		x		MEE^n^	49	20 (41%)^ǂ^	7	5					[18]
India	F^l^				x	x		101	1 (1%)	1						[46]
USA	F^l^				x			120	5 (4%)							[47]
Spain	R^r^					x		311	41 (13%)	15	25			1		[17]
Czech Republic	F^l^	x	x	x	x			1156	36 (3%)	19					1	[26]
Thailand	F^l^	x	x	x		x		151	17 (11%)	4	9	3		1		[19]
Iran	R^r^	x	x		x	x		151	14 (9%)		12			1		[27]

^a^sampling method; ^b^culture on trypticase-soy agar; ^c^microscopic detection of *Brachyspira; *^d^biochemical differentiation of *Brachyspira*: indol production, hippurate-cleavage, and α-galactosidase and ß-glucosidase activity; ^e^polymerase chain reaction; ^f^sequencing; ^g^*Brachyspira pilosicoli; *^h^*Brachyspira innocens; *^i^*Brachyspira intermedia; *^k^*Brachyspira hyodysenteriae; *^l^fecal sample; ^m^culture on casein-soybean flour-peptone agar with 10% ovine blood + spectinomycin; ^n^multilocus enzyme electrophoresis; ^o^ribotyping; ^r^rectal swab; ^s^pulsed-field gel electrophoresis; *sampling bias: 29 laboratory Beagle dogs tested after previous diarrhea outbreak, 17 pet dogs with diarrhea; ^ǂ^sampling bias: pet shop puppies originating from breeding kennels

The genus *Brachyspira* consists of nine officially named species that have been isolated from different animal species as well as humans [5]. *Brachyspira (B.) hyodysenteriae*, *B. hampsonii*, and *B. suanatina* cause dysentery in pigs and is associated with severe mucohemorrhagic colitis [6]. *B. pilosicoli* is the etiological agent of porcine intestinal spirochetosis [7, 8], characterized by persistent, mild, mucoid, and non-hemorrhagic diarrhea in addition to a reduced daily weight gain [9]. *B. pilosicoli* has been shown to also infect several other species including chickens, dogs, wild rodents, and humans [10–12]. In chickens, infection with *B. pilosicoli* leads to persisting but mild, non-hemorrhagic diarrhea and, most importantly, reduced egg production [13]. Aside from *B. pilosicoli*, avian spirochetosis is also caused by *B. intermedia* and *B. alvinipulli* [14, 15].

Although occasionally isolated (Table 1), the role of *B. pilosicoli* as a potential pathogen in dogs has not yet been determined. A case report demonstrated *B. pilosicoli* isolation in combination with mucohemorrhagic diarrhea in three dogs in Japan [16]. Another investigation also found a significant association between the shedding of *B. pilosicoli* and diarrhea in dogs [17], whereas this link was not observed in other studies [18–20].

A zoonotic potential of *B. pilosicoli* has been suggested based on comparative analyses of *B. pilosicoli* isolates from several different species, including dogs and humans [21]. Close geographical proximity combined with closely related electrophoretic types of isolated strains as characterized by multilocus enzyme electrophoresis (MEE) indicates that the colonization of several different animals, including dogs, with *B. pilosicoli* is a potential risk factor for zoonotic infections [21, 22].

Dogs are kept in varying housing conditions and may participate in specific activities or work, such as hunting, which could be associated with the rate of enteropathogen shedding. We hypothesized that these lifestyle and environmental factors have an impact on the transmission and colonization of *Brachyspira* in dogs. Thus, we aimed to (i) identify dogs infected with the zoonotic agent *B. pilosicoli* and patients with canine spirochetosis, which is currently a controversial disease, and (ii) explore potential risk factors for *Brachyspira* shedding in dogs, such as age, breed, housing type, feeding regimen, prior antibiotic treatment, outdoor activities, and hunting.

## Results

### Optimization of cultural detection of *Brachyspira* in canine feces

*Brachyspira* were isolated from 29 of the 275 investigated fecal or rectal swab samples. Samples from 3 dogs allowed the isolation of *Brachyspira* spp. on selective FAA, with no growth on the selective TSA (Table 2). In 4 cases, *Brachyspira* only grew on selective TSA, whereas selective FAA remained clear. The remaining 20 *Brachyspira* isolates showed growth on both selective agar types. We conclude that cultural investigation of canine fecal samples using selective FAA and TAA increases the sensitivity to detect *Brachyspira* spp.

**Table 2:** Differentiation of *Brachyspira (B.)* spp. following cultural isolation from rectal swabs or fecal samples. This table summarizes the results for the differentiation methods applied in the study

Sample ID	Culture		MALDI-TOF^c^ (score)	MP-PCR^d^			Nox^e^ sequencing	16 S rRNA^h^ sequencing
	TSA^a^	FAA^b^		Nox^e^	abgB^f^	tnaA^g^		
23	+^#^		NOIP^i^	+^#^	-^ф^	-^ф^	*B. canis*	*B. canis*
26	+^#^		*B. pilosicoli* (2.29)	+^#^	+^#^	-^ф^	*B. pilosicoli*	*B. pilosicoli*
40	+^#^		NOIP^i^	+^#^	-^ф^	-^ф^	*B. canis*	*B. canis*
88	+^#^		NOIP^i^	+^#^	-^ф^	-^ф^	*B. pulli*	*B. pulli/murdochii/intermedia*
96	+^#^	+^#^	*B. murdochii* (1.97)	+^#^	-^ф^	+^#^	*B. intermedia* ^*¥*^	*B. intermedia/hampsonii/hyodysenteriae*
102	+^#^	+^#^	NOIP^i^	+^#^	-^ф^	-^ф^	*B. canis*	*B. canis*
108	+^#^	+^#^	NOIP^i^	+^#^	-^ф^	-^ф^	*B. pulli*	*B. pilosicoli/muris/murdochii/intermedia*
175	+^#^	+^#^	NOIP^i^	+^#^	-^ф^	-^ф^	*B. canis*	*B. canis*
177	+^#^	+^#^	NOIP^i^	+^#^	-^ф^	-^ф^	*B. canis*	*B. canis*
178	+^#^	+^#^	NOIP^i^	+^#^	-^ф^	-^ф^	*B. pulli*	*B. pulli/murdochii/intermedia*
179	+^#^	+^#^	NOIP^i^	+^#^	-^ф^	-^ф^	*B. canis*	*B. canis*
184	-^ф^	+^#^	NOIP^i^	+^#^	-^ф^	-^ф^	*B. pulli*	*B. pulli/murdochii/intermedia*
283*	-^ф^	+^#^	NOIP^i^	+^#^	-^ф^	-^ф^	*B. canis*	*B. canis*
283.1*	+^#^	-^ф^	NOIP^i^	+^#^	-^ф^	-^ф^	*B. canis*	*B. canis*
283.2*	+^#^	+^#^	NOIP^i^	+^#^	-^ф^	-^ф^	*B. canis*	*B. canis*
283.3*	+^#^	+^#^	NOIP^i^	+^#^	-^ф^	-^ф^	*B. canis*	*B. canis*
284	+^#^	+^#^	NOIP^i^	+^#^	-^ф^	-^ф^	*B. canis*	*B. canis*
287	+^#^	-^ф^	NOIP^i^	+^#^	-^ф^	-^ф^	*B. canis*	*B. canis*
288	+^#^	+^#^	NOIP^i^	+^#^	-^ф^	-^ф^	*B. canis*	
289	+^#^	+^#^	NOIP^i^	+^#^	-^ф^	-^ф^	*B. canis*	*B. canis*
293	+^#^	+^#^	NOIP^i^	+^#^	-^ф^	-^ф^	*B. canis*	*B. canis*
302	+^#^	-^ф^	NOIP^i^	+^#^	-^ф^	-^ф^	*B. canis*	
303	+^#^	-^ф^	NOIP^i^	+^#^	-^ф^	-^ф^	*B. pulli*	*B. pulli/sp.*
313	+^#^	+^#^	NOIP^i^	+^#^	-^ф^	-^ф^	*B. canis*	*B. canis*
330	+^#^	+^#^	NOIP^i^	+^#^	-^ф^	-^ф^	*B. canis*	*B. canis*
346	+^#^	+^#^	NOIP^i^	+^#^	-^ф^	-^ф^	*B. pulli*	*B. pulli/murdochii/intermedia*
352/7^ǂ^	+^#^	+^#^	*B. pilosicoli* (2.06)	+^#^	+^#^	-^ф^	*B. pilosicoli*	*B. pilosicoli*
352/8^ǂ^	+^#^	+^#^	*B. pilosicoli* (2.08)	+^#^	+^#^	-^ф^	*B. pilosicoli*	*B. pilosicoli*
352/9^ǂ^	+^#^	+^#^	*B. pilosicoli* (2.27)	+^#^	+^#^	-^ф^	*B. pilosicoli*	*B. pilosicoli*

^a^trypticase soy agar; ^b^fastidious anaerobe agar; ^c^matrix-assisted laser desorption/ionization – time of flight; ^*d*^multiplex polymerase chain reaction; ^e^NADH oxidase gene; ^f^hippurate-hydrolase gene; ^g^tryptophanase gene; ^h^ribosomal ribonucleic acid; ^i^NOIP - no organism identification possible; *sample 283 was isolated from the mother of three puppies from which samples 283.1, 283.2, and 283.3 were obtained; ^**ǂ**^samples 352/7, 352/8, and 352/9 originated from dogs housed in the same kennel (pack of Beagle dogs); ^#^indicates a positive result (either culture growth or banding in the MP-PCR); ^ф^indicates a negative result (either no culture growth or no banding in the MP-PCR); ^*¥*^no good quality sequence could be obtained for isolate no. 96, putative result

### Longitudinal detection of *Brachyspira* in a colony of Beagle dogs

**Fig. 1:**
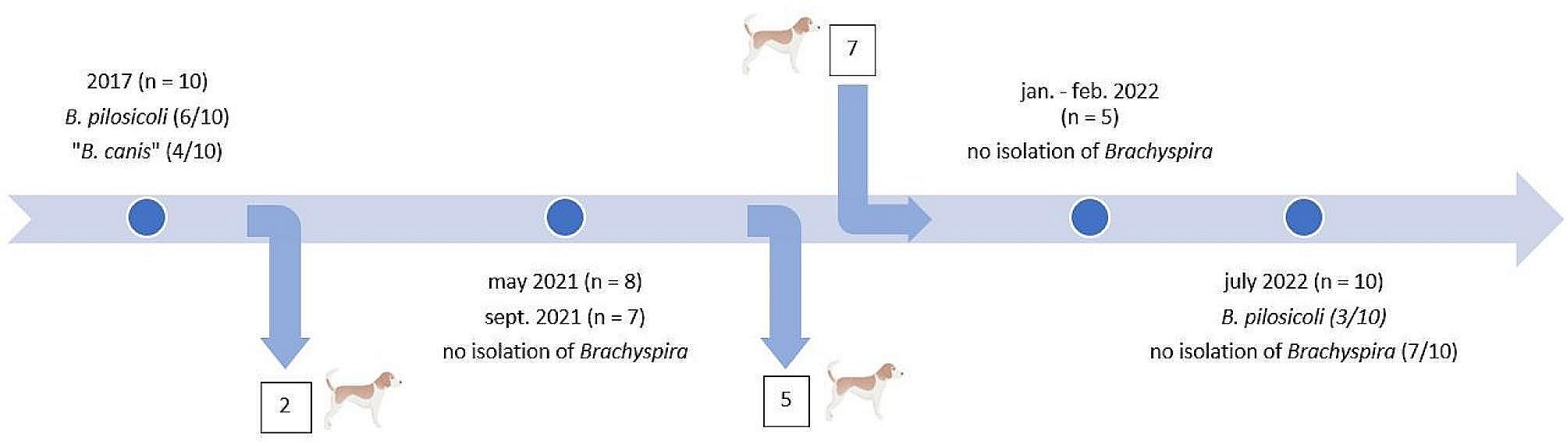
Timeline summarizing the sampling of the Beagle colony between June 2017 and July 2022. Events (blue dots on the timeline) indicate sampling via rectal swaps or fecal samples and investigation for *Brachyspira* by culture. Arrows pointing away from or towards the timeline indicate dogs (numbers in boxes) permanently leaving or joining the pack

**Fig. 2:**
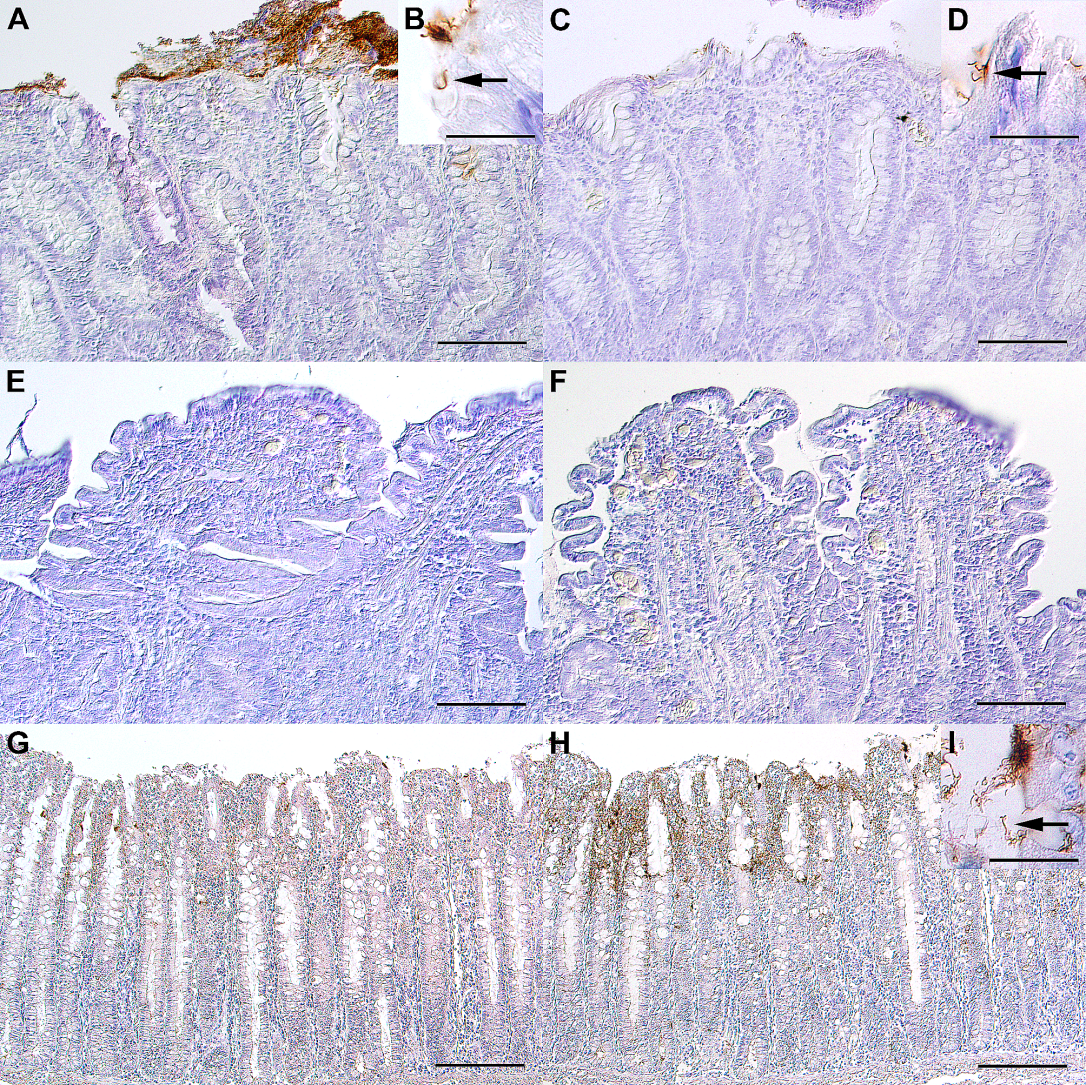
Immunohistochemistry for “*B. canis”* (**A, B, E, G**) and *B. pilosicoli* (**C, D, F, H, I**) in canine colon (**A-D**), canine duodenum (**E, F**) and porcine colon (**G-I**). **A**: Moderate numbers of “*B. canis”*-immunoreactive bacteria on the luminal surface of the colon. **B**: Higher magnification from **A** showing the characteristic spiral-shaped morphology of *Brachyspira* (arrow). **C**: Minimal numbers of *B. pilosicoli*-immunoreactive bacteria on the luminal surface of the colon. **D**: Higher magnification from **B** showing the characteristic spiral-shaped morphology of *Brachyspira* (arrow). **E**: No detection of “*B. canis”*-immunoreactive bacteria in the duodenum. **F**: No detection of *B. pilosicoli*-immunoreactive bacteria in the duodenum. **G**: Minimal numbers of “*B. canis”*-immunoreactive, spiral-shaped bacteria on the luminal surface of the colon of a pig with intestinal spirochetosis associated with the detection of *B. pilosicoli* by PCR. **H**: Large numbers of *B. pilosicoli*-immunoreactive, spiral-shaped bacteria on the luminal surface of the colon of a pig with intestinal spirochetosis associated with the detection of *B. pilosicoli* by PCR. **I**: Higher magnification from **H** showing the characteristic spiral-shaped morphology of *Brachyspira* (arrow). Immunohistochemistry employing polyclonal rabbit anti-“*B. canis”-*antibody or rabbit anti-*B. pilosicoli*-antibody and the avidin-biotin-peroxidase complex method with DAB chromogen and hematoxylin counterstain. Differential interference contrast microscopy. Bars **A, C, E, F** = 100 μm; **B, D, I** = 20 μm; **G, H** = 200 μm

In a preliminary experiment in 2017, a colony of 10 Beagle dogs was investigated for *Brachyspira* shedding. *Brachyspira* and *B. pilosicoli* were culturally detected and identified via MALDI-TOF MS and multiplex-PCR (MP-PCR) in 10 and 6 dogs, respectively. Sequencing of the *nox* gene confirmed *B. pilosicoli* in 6 and suggested “*B. canis”* for the remaining 4 isolates. Repeated sampling of 8 dogs in May and September of 2021 (Fig. 1) did not result in any *Brachyspira* being isolated. Investigation of rectal swabs obtained from all dogs of the newly arranged pack (n = 10) in September 2022 (Fig. 1) resulted in the isolation of *Brachyspira* from 3 dogs. MALDI-TOF MS, MP-PCR, and sequencing of the *nox* and *16s rRNA* genes identified 3 isolates as *B. pilosicoli*. Two of these 3 dogs had been positive for *B. pilosicoli* in 2017, while 1 isolate originated from a dog that had only recently joined the pack. These results indicate that the investigated kennel has repeatedly been infected or colonized with *B. pilosicoli* and “*B. canis*”. With the collection of each rectal swab, pertinent clinical signs were recorded for each dog. Diarrhea or other clinical signs of gastrointestinal disease were not documented at any of the time points of sampling. Additionally, gastrointestinal signs, abnormal physical examination findings, or clinicopathologic changes were not documented during any of the annual health checks of the dogs. However, in October of 2022, one of the dogs that had twice tested positive for *B. pilosicoli* developed chronic gastrointestinal signs (diarrhea, melena, inappetence, vomiting, and mild body weight loss). This led to the dog being admitted to the Internal Medicine service, Department for Small Animals at the University of Leipzig Faculty of Veterinary Medicine (UL-FVM) for diagnostic investigation. Over the course of several months, beginning in October of 2022, this dog continued to show gastrointestinal signs with progressive weight loss. In addition to routine clinicopathologic investigation and diagnostic imaging, an upper and lower gastrointestinal endoscopy was performed and gastric, duodenal, and colonic biopsies were obtained (ileum could not be intubated) and histopathologically investigated. Mild lymphoplasmacellular enteritis with structural changes (shortened villi, dilated lacteals, and dilated crypts with focal crypt abscesses) and mild lymphoplasmacellular colitis were recorded. Integrating the results of bloodwork, urinalysis, fecal parasitology, abdominal ultrasonography, and gastrointestinal endoscopy led to the diagnosis of an inflammatory protein-losing enteropathy (PLE). The dog had marked hypoalbuminemia (16 g/L, reference interval [RI]: 26–40 g/L), hypocholesterolemia (2.42 mmol/L, RI: 2.87–8.07 mmol/L), normal urine protein/creatinine ratio (0.2, RI: <0.5), unremarkable serum bile acid stimulation test (preprandial bile acid concentration: 13.5 µmol/L, RI: 0–20 µmol/L; postprandial bile acid concentration: 0.7 µmol/L; 0–40 µmol/L), unremarkable adrenal response (baseline cortisol concentration: 37.3 nmol/L; ACTH-stimulated cortisol concentration: 322.9 nmol/L), hypocobalaminemia (139 pmol/L; RI: 173–599 pmol/L), and hypofolatemia (18.5 nmol/L; RI: 21.1–54.0 nmol/L). Abdominal ultrasonography revealed mild mesenteric lymphadenomegaly and multifocal irregularities of the small intestinal wall with reduced distinction of the wall layering and hyperechoic mucosal striations. Duodenal and colonic biopsies from the dog were evaluated by immunohistochemistry, which confirmed positive staining for *Brachyspira* on the luminal surface of the colon (Fig. 2A-D). However, the signal was substantially stronger with the rabbit anti-“*B. canis*”-antibody (Fig. 2A and B) compared to the rabbit anti-*B. pilosicoli* antibody (Fig. 2C and D), which was unexpected given the cultural detection of *B. pilosicoli* in rectal swabs from this dog. There was no positive staining with either antibody in the duodenal biopsies from this dog (Fig. 2E and F).

### Association of *Brachyspira* detection with environmental and lifestyle factors

Despite the high detection rate of *B. pilosicoli* in the described kennel, it was unknown whether *B. pilosicoli* is commonly shed in Germany and if shedding is associated with specific environmental and lifestyle factors, such as shelter or kennel origin. *Brachyspira* spp. were isolated in 26 (11%) of all 235 dogs included in the statistical analyses (Table 2). Isolation was significantly more frequent from dogs less than 1 year of age than from older dogs (*P* = 0.0247), and this remained significant in the multivariate analysis (*P* = 0.0140); (Table 3).

**Table 3:** Association of Brachyspira isolation rates with the dogs’ signalment, demographic variables, medical and diet history, and current health status

Variable	Brachyspira isolated	Brachyspira not isolated	P_univariate_	P_multivariate_
Patient characteristics				
Age < 1 year	8/26 (31%)	26/206 (13%)	**0.0247**	**0.0140**
Male gender	9/23 (39%)	110/205 (54%)	0.1852	
Neutered/spayed	10/26 (39%)	62/200 (31%)	0.4488	
Mixed breed	18/26 (69%)	61/201 (30%)	**0.0001**	0.3283
Body weight				
< 10 kg	3/8 (37%)	44/174 (25%)	0.6325	
10–25 kg	4/8 (50%)	88/174 (51%)		
> 25 kg	1/8 (13%)	42/174 (24%)		
BCS < 5 (scale of 9)	1/2 (50%)	15/57 (26%)	0.4845	
Environmental & lifestyle factors				
Shelter origin	17/26 (65%)	43/209 (21%)	**< 0.0001**	**< 0.0001**
Hunting dog	2/26 (8%)	84/206 (41%)	**0.0003**	0.0531
Kennel dog	17/26 (65%)	77/202 (38%)	**0.0083**	0.0706
Leashed walks	15/24 (63%)	155/197 (79%)	0.0909	
Travel/stay abroad	10/19 (53%)	68/192 (35%)	0.1452	
Medical and diet history				
Prior antibiotics	9/24 (38%)	60/198 (30%)	0.4783	
Pre-or probiotics	1/20 (5%)	18/171 (11%)	0.3963	
Vaccination	24/26 (92%)	186/204 (91%)	0.9847	
Deworming	25/26 (96%)	168/200 (84%)	0.0589	
Anti-inflammatories	4/18 (22%)	45/175 (26%)	0.7426	
NSAID	2/18 (11%)	27/175 (15%)	0.6129	
Steroid	2/18 (11%)	8/175 (5%)	0.2893	
Raw feeding (BARF diet)	1/21 (5%)	10/175 (6%)	0.8546	
Recent diet change	4/14 (29%)	43/156 (28%)	0.9359	
Kibble diet	19/21 (91%)	150/167 (90%)	0.9246	
Wet/canned diet	11/21 (52%)	79/167 (47%)	0.6609	
Commercial diet	22/23 (96%)	174/184 (95%)	0.8219	
Home-made diet	2/23 (9%)	44/184 (24%)	0.0701	
Dietary supplements	4/23 (17%)	25/184 (14%)	0.6295	
Co-infection with Giardia and/or Cryptosporidium	0/2 (0%)	7/19 (37%)	0.1890	
Clinical signs of gastrointestinal disease				
Known or suspected chronic enteropathy	4/25 (16%)	33/187 (18%)	0.8370	
Defecation frequency*	1 (0–2)	1 (0–3)	0.7564	
Fecal consistency AHDi*	0.5 (0–2.5)	0 (0–3)	0.8041	
Waltham feces score	3.5 (1.5–4)	4 (1–4.5)	0.7874	
Hematochezia/melena	0/26 (0%)	11/190 (6%)	0.0885	
Mucus	2/26 (8%)	19/188 (10%)	0.6893	
Undigested material	0/26 (0%)	9/185 (5%)	0.1196	
Flatulence	1/13 (8%)	32/163 (20%)	0.2431	
Abdominal pain	3/14 (21%)	13/166 (8%)	0.1328	
Weight loss	2/25 (8%)	16/183 (9%)	0.9003	
Vomiting	3/25 (12%)	15/172 (9%)	0.6516	
Vomiting score*	0 (0–1)	0 (0–1)	0.6518	
Clinical severity score*	1.5 (0–4.5)	1 (0–7)	0.5415	
At least one GI sign^‡^	15/25 (60%)	105/195 (54%)	0.5592	

*criterion or criteria of the acute hemorrhagic diarrhea index (AHDi; [24]); ‡includes diarrhea (and/or hematochezia/melena, mucus, other material), vomiting, weight loss, abdominal pain, and/or flatulence

In 3 of the 5 shelters, *Brachyspira* spp. were isolated from several (5/12, 9/14, and 3/17) dogs. *Brachyspira* spp. were not detected in the other 2 shelters (0/4 and 0/13 dogs). Being housed at a shelter was a significant risk factor for the isolation of *Brachyspira* spp. (*P* < 0.0001), and this association remained significant in the multivariate analysis (*P* < 0.0001). *Brachyspira* spp. were also significantly more frequently isolated from kennel-housed dogs (*P* = 0.0083), but kennel housing did not remain a significant risk factor in the multivariate model (*P* = 0.0706). Kennel dogs included shelter dogs and some pet dogs that are occasionally held in kennels (often single or double dog kennels) alternating with time spent in the home of the owners (mostly hunting dogs).

Univariate analysis further identified mixed-breed dogs to have a higher prevalence of *Brachyspira* than pure-bred dogs (*P* = 0.0001) and being used as a hunting dog to be a protective factor for *Brachyspira* isolation (*P* = 0.0003). However, both associations did also not remain statistically significant in the multivariate model (*P* = 0.3283 and *P* = 0.0531).

*Brachyspira sp.* isolation was not significantly associated with any other patient characteristic, environmental or lifestyle factors, medical or diet history criteria, or clinical signs of gastrointestinal disease. Specifically, the isolation of *Brachyspira* sp. was not correlated with the presence or severity of vomiting or diarrhea as assessed by AHDi and Waltham score, the presence of flatulence, abdominal pain, or weight loss, overall clinical severity score, nor co-infection with *Giardia* sp. and/or *Cryptosporidium* sp. (all *P* > 0.05; Table 3).

As *B. pilosicoli* was isolated from only 1 dog, statistical analyses on the prevalence or risk factors for *B. pilosicoli* isolation were not possible.

Statistical analyses of the correlation between the isolation of “*B. canis*” and possible risk factors did not vary from the general association with the isolation of *Brachyspira* spp. (Table 3 and Suppl. Table 1). We found a statistically significant association between travel/a stay abroad of the dog and the isolation of “*B. pulli*” (*P* = 0.0173), which was also confirmed in the multivariate model (*P* = 0.0169; Suppl. Table 1).

### Sequencing of the *nox* and *16 S rRNA* genes to differentiate canine *Brachyspira* isolates

After confirmation of *Brachyspira* species by MP-PCR, the isolates were further differentiated by nox and 16 S rRNA gene sequencing. BLAST (basic local alignment search tool) analyses of the partial *nox* sequence identified 18 isolates as “*B. canis”* (16 isolates with 100% identity and 2 isolates with an identity of 99.8%), 6 isolates as “*B. pulli”* (5 isolates with 96.4% identity and 1 isolate with an identity of 96.1%), and 4 isolates as *B. pilosicoli* (100% identity). Despite repeated sequencing, a good-quality sequence could not be obtained for isolate no. 96, putatively identified as *B. intermedia* (identity 94.0%). As isolate no. 96 showed an amplification product for the *tnaA* gene in the MP-PCR, identification as *B. intermedia* seems likely, because *B. hyodysenteriae* and *B. suanatina* are not expected to be isolated from canine samples. BLAST analyses of the *16 S rRNA* gene identified 16 isolates as “*B. canis”* (100% identity) and 4 isolates as *B. pilosicoli* (100% identity). Isolates identified as “*B. pulli”* by sequencing of the *nox* gene were not unequivocally identified by *16 S rRNA* sequencing as high percentages of identity were seen with several different *Brachyspira* species (Table 2). Isolates no. 288 and 302, identified as “*B. canis*” by sequencing of the *nox* gene, could not be successfully examined via *16 S rRNA* sequencing. In conclusion, identification of *B. pilosicoli* isolated from canine feces is possible by different methods, but differentiation of “*B. canis”* as well as *“B. pulli”* remains challenging though nox sequencing appears to be a suitable approach.

## Discussion

The results of our study demonstrate that the investigated cohort of dogs originating from the geographic area of central Germany shed different *Brachyspira* spp. and that “*B. canis*” and “*B. pulli*” appear to be the most common *Brachyspira* spp. whereas shedding of *B. pilosicoli* was only observed in 1 of the 235 dogs sampled in this study apart from the Beagle pack. This cohort is not a true representative of the dog population of central Germany as it is geographically not equally distributed. However, we deliberately investigated a diverse group of dogs to identify risk factors for spirochetosis. Our data reveal an association between shedding Brachyspira and dogs being housed at animal shelters. Though this association needs confirmation for the enteric pathogen B. pilosicoli, this finding indicates that the form of husbandry plays an important role in spirochetes circulating among dogs. The longitudinal investigation of the Beagle colony also shows that *B. pilosicoli* can infect a number of dogs within a single group and that shedding of *B. pilosicoli* might undergo a recurrent course in a dog colony. Under such circumstances, the risk of infection – including zoonotic transmission – is most likely substantially increased.

We also putatively detected *B. intermedia* in 1 dog. Though *nox* sequencing did not allow unambiguous identification of *B. intermedia*, detection of *tna*A in the MP-PCR in this canine isolate is highly suggestive of *B. intermedia*. *B. hyodysenteriae* and *B. suanatina* have not been reported in dogs (Table 1), but can not be entirely excluded. This example shows that combining different diagnostic methods increases the likelihood of successful differentiation of *Brachyspira* species, which remains challenging for routine diagnostic laboratories. *B. intermedia* is a common bacteria in the colon of poultry, and isolation of *B. intermedia* from dog feces may be due to the prior intake of contaminated chicken meat or chicken intestine [23–25].

*B. pilosicoli*, as the agent suspected to cause canine intestinal spirochetosis, was isolated from only 1 dog, aside from the *B. pilosicoli* isolates originating from the colony of Beagle dogs described. The low detection rate of the zoonotic pathogen *B. pilosicoli* in the tested dog population suggests a low public health risk for human intestinal spirochetosis emanating from dogs in the geographic area investigated (i.e., central Germany). To accommodate the difficulty of isolating Brachyspira in pure culture, we used two selective agars and concluded that combining selective TSA and selective FAA increases the sensitivity of culturing *Brachyspira* from canine fecal and rectal swab samples.

In previous studies, *B. pilosicoli* [26] and *Brachyspira* spp [19, 27]. were more frequently detected in dogs that were younger than 1 year. Being under the age of 1 year was also a significant risk factor for shedding *Brachyspira* in the dog population reported here. The prevalence of *Brachyspira* isolation from fecal samples and rectal swabs obtained from dogs varies between 1 and 19% in different studies (Table 1), agreeing with the 11% isolation rate in this study. The varying prevalences may be due to local differences in the dog populations (e.g., genetics, environmental factors, epigenetic effects). Another variable is the geographic origin of the dogs in different studies. Similar to our findings, laboratory Beagles and kennel dogs had a higher prevalence of *Brachyspira* isolation than companion animals in other studies, with isolation rates up to 52% [18, 20]. Another study also identified the origin of dogs as relevant and found that dogs from animal shelters had a higher risk of shedding *B. pilosicoli* [17]. Our study agrees with these findings, as the shedding of *Brachyspira* was significantly higher in dogs that are housed in animal shelters, although we did not isolate *B. pilosicoli* from any of the shelter dogs. We also found a slightly but not significantly lower *Brachyspira* detection rate in hunting dogs compared to companion animals, which confirms the previous observation that use as a hunting or guard dog is not linked to a higher risk of *Brachyspira* shedding in feces [17]. This is a rather surprising result given that hunting dogs come in close contact with different wild animals, including rodents from which different *Brachyspira* species (e.g., *B. pilosicoli*) have been isolated [12] and that pose a potential risk for *Brachyspira* colonization or infection in hunting dogs.

Some *Brachyspira* species are regarded as apathogenic as their detection is generally not associated with diarrhea, and experimental infection has failed to induce clinical signs of disease. Further, despite treatment with dexamethasone for immune suppression, pigs did not develop clinical disease after application of *B. innocens*, *B. murdochii*, or *B. intermedia* [28]. Although “*B. canis*” and “*B. pulli*” are also generally considered to be commensals [21], their role as pathogens is not completely excluded, and a possible association between diarrhea and isolation of “*B. pulli”* has been documented [19]. Thus, to identify potential risk factors only associated with the isolation of 1 of both *Brachyspira* spp., the statistical analyses were also performed separately for “*B. canis*” and “*B. pulli*”. Statistical analyses of the correlation between the isolation of “*B. canis*” and the possible risk factors investigated did not differ from the associations with the general isolation of *Brachyspira* (Table 3 and Suppl. Table 1). This could be explained by the fact that most of the isolated *Brachyspira* spp. were identified as “*B. canis*”. Consequently, age less than 1 year and shelter origin of the dogs and samples were also risk factors for the shedding of “*B. canis*”. In contrast, statistical analyses of the association between “*B. pulli”* and the possible risk factors revealed a different result. Our analysis showed a statistically significant association between travel/a stay abroad of the dog and the isolation of “*B. pulli*”. Similar to a previous study [17], we detected “*B. canis*” and “*B. pulli*” in a number of fecal samples or rectal swabs from apparently healthy dogs and did not document an association with diarrhea. This agrees with the classification of “*B. canis*” and “*B. pulli*” as apathogenic [18, 21]. Thus, the proposed pathogenic potential of “*B. pulli”* [19] could not be verified in our study based on the risk factors analyzed, although statistical power was limited due to the small number of isolated *B. pulli* in our study cohort.

*B. pilosicoli* causes intestinal spirochetosis in pigs and poultry, as demonstrated by experimental infections [7, 11, 13]. Its role as an intestinal pathogen in dogs is currently less clear, as an association with diarrhea is inconsistent across studies [16–20] and an experimental infection of dogs has not been reported. Thus, further clarification of the pathogenicity of *B. pilosicoli* is needed. Here, we describe the 5-year history of a Beagle colony that was shown to shed *B. pilosicoli* in 2017 and 2022. Despite the investigation of a large number of samples, *B. pilosicoli* was not detected in this colony in 2021. As the prevalence of *B. pilosicoli* infection in dogs appears generally low in central Germany, we speculate that this colony was not reinfected but that the introduction of the 7 young dogs in 2022 enhanced the shedding and transmission of this pathogen within the colony. A direction for future research would be strain typing of the different isolates that were recovered from this colony over the past years to ascertain whether these strains have persisted. Additionally, these strains should be examined for potential antibiotic-resistance genes, especially as this shelter is located in close proximity to a large animal clinic. Of note, this colony does not have a history of increased incidences of diarrhea and isolates of *B. pilosicoli* did not originate from diarrheic feces. This shows that the zoonotic pathogen *B. pilosicoli* can be shed from dogs that are clinically apparently healthy. Interestingly, one of the dogs that tested positive for B. pilosicoli in 2017 and again in 2022 developed chronic signs of gastrointestinal disease (diarrhea, melena, inappetence, vomiting, and weight loss) later in 2022. This case could support the hypothesis that canine spirochetosis caused by *B. pilosicoli* can be asymptomatic and that shedding can be triggered by external and/or internal factors (e.g., increased stress with the introduction of new members in the pack). Co-infection of this dog with “*B. canis*” based on immunohistologic detection agrees with previous reports, demonstrating the isolation of multiple *Brachyspira* spp. from individual animals. This has been also shown for poultry [29, 30] and pigs [31, 32]. Hematological findings, blood biochemistry, urinalysis, diagnostic imaging, and histologic documentation of lymphoplasmacellular enteritis with structural lesions and colitis indicate an inflammatory PLE in this dog. Enteritis is an unexpected finding in spirochetosis cases as *B. pilosicoli* targets the large intestine, though the ileum is also infected by this agent in poultry [33]. Overall, this case of a PLE exemplifies the difficulty of confirming or excluding *B. pilosicoli* as an etiology of disease in dogs.

To our knowledge, this is the first study suggesting that specific anti-*Brachyspira* antisera allow differentiation of *Brachyspira* spp. via immunohistochemistry. Due to the close genetic relationship and the high likelihood of cross-reacting antigens in both *Brachyspira* spp., purification of specific antibodies via preadsorption is considered to be crucial. In pigs, cross-reacting antibodies against *B. hyodysenteriae* and *B. pilosicoli* have been recorded [34]. Immunohistochemistry with specific anti-*Brachyspira* sp.-antibodies may also be a possible differentiation method for *Brachyspira* spp. such as *B. hyodysenteriae*, *B. pilosicoli*, *B. suanatina* and *B. innocens* in swine and the diagnosis of swine dysentery.

## Methods

### Ethics approval and informed owner consent

Ethics approval was granted for the study by the local ethics committee at the University of Leipzig Faculty of Veterinary Medicine (UL-FVM Ethics Committee; approval# EK9-2021, approved 05-31-2021), and written informed consent was obtained from each owner or caretaker prior to enrollment of a dog. A standard study questionnaire (Suppl. File S1) was completed by the owners or caretakers of enrolled dogs to obtain information about the dogs’ signalment (including age, sex, neuter status, body weight, body condition score [BCS], and breed), demographic data (diet, housing conditions, outdoor activities, hunting, and travel history), and health status (including deworming and vaccination status) with special emphasis on clinical signs of gastrointestinal conditions (presence and severity of vomiting and/or diarrhea as assessed by the acute hemorrhagic diarrhea index [AHDi] [35] or Waltham feces score [36], weight loss, abdominal pain, and flatulence) and prior antimicrobial, probiotic, and/or prebiotic administration.

### Sampling population and sample collection

**Fig. 3:**
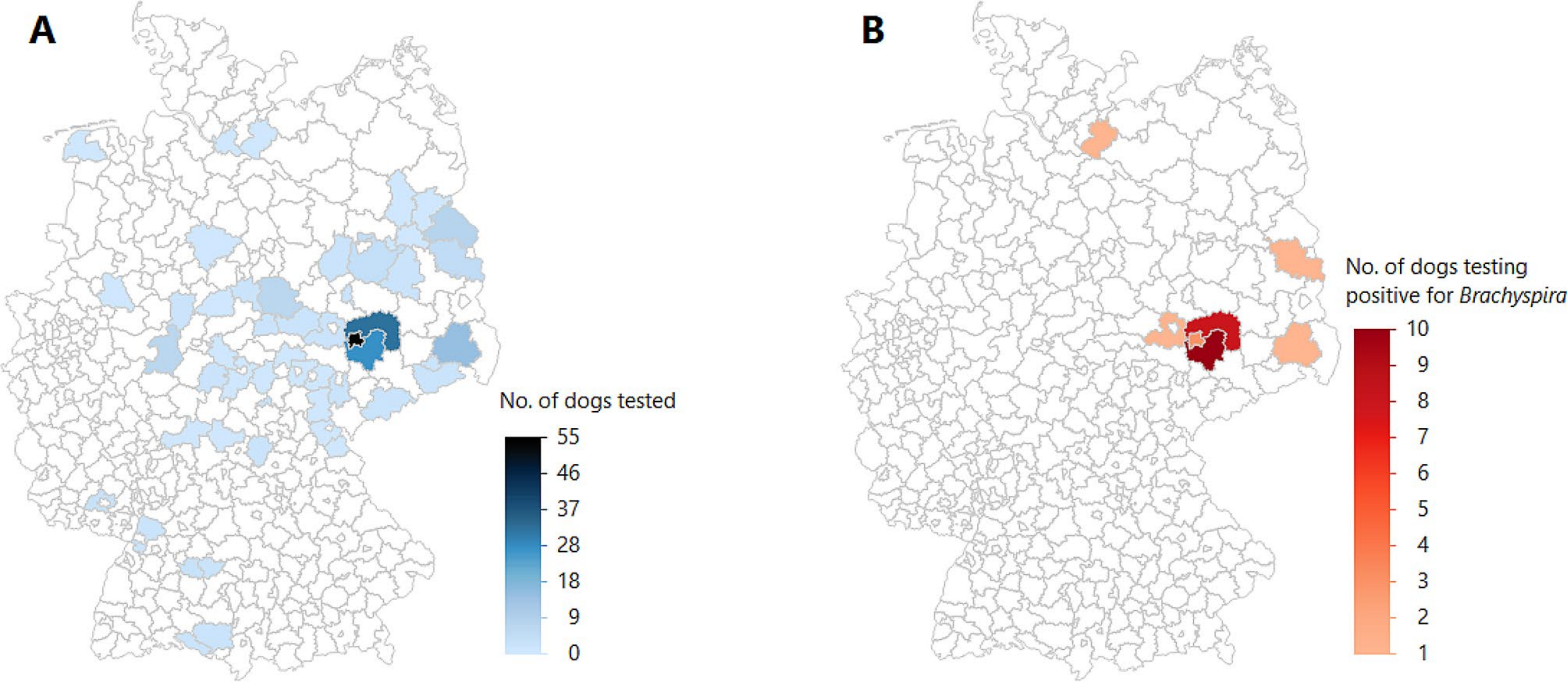
**A**: Geographic origin of the dogs included in the sampling of an independent cohort, localized to the respective administrative districts within the Federal Republic of Germany. **B**: Quantitative geographical visualization (by administrative districts) of the origin of dogs testing positive for *Brachyspira*

*Sampling of an independent cohort of dogs* – Between May 2021 and August 2022, rectal swabs (*n* = 216) and fecal samples (*n* = 19) were collected from 235 dogs, including 17 research kennel-housed dogs and 218 privately owned pet and/or working dogs. These dogs were recruited at the Department for Small Animals at the UL-FVM, five different veterinary offices in the greater Leipzig area (Germany), five small animal shelters in the central part of Germany, three hunting events (two in the federal state of Saxony and one in the federal state of Brandenburg, Germany), and from personal contacts (Fig. 3). All samples were refrigerated immediately after collection until further processing, and the samples were cultured within 24 h after collection.

*Longitudinal sampling of a Beagle colony* – Rectal swabs (*n* = 35) and fecal samples (*n* = 5) were collected between June 2017 and July 2022 from a total of 17 Beagle dogs housed in close contact within a research kennel. Dogs generally live in this kennel until they are about 8 years of age, after which they are adopted out as pet dogs. The animals were treated in accordance with the principles outlined in the EU Directive 2010/63/EU, the European Convention for the Protection of Vertebrate Animals Used for Experimental and other Scientific Purposes, and the German Animal Protection Law (*Tierschutzgesetz*). After the first sampling of ten dogs in 2017, two dogs left the colony and were no longer available for re-testing in 2021. In January of 2022, another five dogs left the pack, and seven new dogs were integrated from a different kennel. *Brachyspira* colonization status was determined via rectal swabs, which were obtained from 10 Beagles in 2017, from eight dogs in 2021, and from 10 dogs in 2022 (Fig. 1). Because of the fluctuation of dogs within the colony, some dogs were tested for *Brachyspira* via rectal swab or fecal sample multiple times, whereas other dogs were sampled only once either in 2017 or in 2022 (Fig. 1). The samples were refrigerated immediately after collection until further processing and were cultured within 24 h after collection.

### Isolation and identification of *Brachyspira*

*Culture* – Culture of *Brachyspira* spp. was performed using a TSA containing 10% horse blood and the following selective antibiotic concentrations (designated as selective TSA): 6.2 µg/mL of colistin, 12.5 µg/mL of rifampicin, and 200 µg/mL of spectinomycin. After using selective TSA for cultivating *Brachyspira* for the first 59 specimens, difficulties in attaining a pure culture arose for one sample due to contamination with anaerobic bacteria other than *Brachyspira*, which also showed a swarming growth pattern. This sample was subsequently streaked on selective fastidious anaerobe agar (FAA; LabM, Lancashire, UK) with 10% equine blood and the antibiotics used in selective TSA, to induce the formation of single colonies and obtain a pure *Brachyspira* culture. Because this modified isolation protocol was successful, all subsequent samples were streaked on both selective TSA and selective FAA to achieve a higher isolation rate.

*Mass spectrometry-based identification of Brachyspira pilosicoli* – Identification of *B. pilosicoli* by MALDI-TOF MS was performed after extraction of samples following a previously published protocol [24]. Importantly, “*B. canis”* and “*B. pulli*” are not represented in the commercial database of the MALDI-TOF MS spectrometer (Microflex LRF, Bruker Daltonics, Bremen, Germany).

**Fig. 4:**
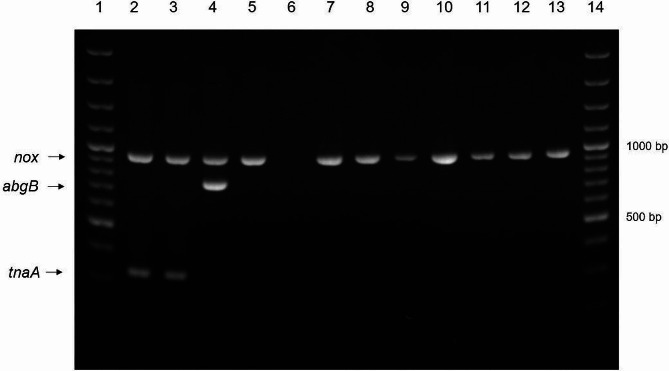
Amplicons generated by multiplex-PCR (MP-PCR) with primers for the following genes: NADH oxidase gene (*nox*; 939 bp amplicon), p-aminobenzoyl-glutamate hydrolase subunit B gene (*abg*B; 744 bp amplicon), and tryptophanase A gene (*tna*A; 325 bp amplicon). Lanes 1 and 14, 100 bp DNA marker; lane 2, *B. hyodysenteriae* strain 404/06; lane 3, *B. intermedia* strain AN26/93; lane 4, *B. pilosicoli* strain 102/06; lane 5, *B. canis* strain S2017; lane 6, negative control; lanes 7–13, isolates no. 102, 108, 175, 177, 178, 179, 184

*Genetic differentiation of Brachyspira* – Detection of the tryptophanase A gene (*tna*A), the p-aminobenzoyl-glutamate hydrolase subunit B gene (*abg*B), and the NADH oxidase gene (*nox*) was conducted via MP-PCR. Generation of the *nox* amplification product in the MP-PCR confirms the genus (Fig. 4), while the *abgB* gene is specific for *B. pilosicoli*. The *tnaA* amplification product is found in *B. intermedia, B. suanatina*, and *B. hyodysenteriae* [24]. Sequencing of the *nox* gene served to differentiate *Brachyspira* as described [24]. The following reference strains were used as positive controls in every MP-PCR analysis: *B. pilosicoli* isolate 102/06 and *B. hyodysenteriae* isolate 404/06 – both from Germany [24], *B. intermedia* isolate AN26/93 from Sweden [37], and “*B. canis”* isolate S2017 from Germany, which was isolated as part of this study. After sequencing the *nox* amplicon, nucleotides 512–926 of the open reading frame were analyzed using the basic local alignment search tool (BLAST) of the National Center for Biotechnology Information (NCBI; conducted between November 2021 and August 2022). *Nox* sequences not showing 100% identity with any published sequence were submitted to GenBank (Suppl. Table 2). Additionally, *Brachyspira* were differentiated by sequencing an amplification product of the *16 S rRNA* gene using the following oligonucleotide primers: kag-011 (reverse) CTTGTGCGGGYYCCCGTC and 584 (forward) CCARACTCCTACGGRAGGC [38–40]. Again, *16 S rRNA* sequences not showing 100% identity with any other published sequence were submitted to GenBank (Suppl. Table 2). Identification of “*B. canis*” was defined as a query cover of 100%, identity of at least 99.76% of the *nox* gene, and 100% identity of the *16 S rRNA* gene sequence to at least 3 sequences deposited by other groups. Identification of “*B. pulli*” was defined as a query cover of at least 99% and identity of at least 96.1% of the *nox* gene to at least 3 sequences deposited by other groups.

### Data and statistical analyses

Data was analyzed for a total of 235 dogs in the independent cohort. Data obtained for the Beagle colony were not included in the analyses for possible risk factors due to selection bias, as these dogs were repeatedly sampled, and the shedding of different *Brachyspira* spp. in this pack was known prior to the start of this investigation. Data analysis was performed using a commercially available statistical software package (JMP^®^ v13.0, SAS Institute, Cary, NC, USA). A Shapiro-Wilk test served to test continuous data for the assumption of normality, based on which the summary statistics were reported as medians (and ranges) for continuous variables; counts (and percentages) were reported for categorical data. Univariate analyses for possible differences or associations were performed using a Wilcoxon rank-sum test (non-parametric two-group comparison of continuous data) or a likelihood ratio or Fisher’s exact test (categorical data with *n* > 5 in all categories or *n* ≤ 5 in at least one category), with statistical significance set at *P* < 0.05. To identify possible covariances and determine true positive or negative risk factors for the isolation of *Brachyspira* sp. from dogs, variables with *P* < 0.05 in univariate analyses were entered in a multivariate stepwise (forward) regression model using the corrected Akaike’s information criterion (*AIC*_*c*_) and a Wald-χ^2^ test.

### Antigen preparation

*B. pilosicoli* antigen was acquired from isolate no. 26, which was cultivated on multiple Columbia blood agar plates under anaerobic conditions. A suspension of the bacteria was obtained by rinsing off the bacterial lawn from each agar plate with 2 mL of phosphate-buffered saline (PBS; pH 7.35). This was similarly done to obtain the “*B. canis”* antigen, using isolate 284.

The bacterial suspensions of *B. pilosicoli* and “*B. canis”* were centrifuged (30 min, 2000× g), the supernatants were discarded, and the remaining pellets were washed twice with PBS. The supernatants were again discarded, and the bacterial pellets were solubilized on a thermal shaker (15 min, 1200 rpm, 37 °C) with a non-ionic detergent-based extraction reagent (B-PER bacterial protein extraction reagent; Thermo Fisher Scientific, Dreieich, Germany) and 5 mM disodium-ethylenediaminetetraacetate (EDTA-Na_2_). After centrifugation (10 min, 16,873× g), the supernatants were carefully separated and subjected to size-exclusion chromatography. The extraction step was repeated for the remaining bacterial pellets.

The extraction reagent was removed using size-exclusion chromatography with a 5 mL-HiTrap-Desalting Column (Merck, Darmstadt, Germany) on the ÄKTAprime plus (Cytiva, Freiburg, Germany). The flow rate was set to 1 mL/min, and the sample (2 mL) was eluted with 10 mL of PBS. The protein-containing fractions were selected at peak optical density (OD_280nm_), pooled, and 5 mM EDTA-Na_2_ and a proteinase inhibitor cocktail (cOmplete EDTA-free tablets; Merck) were added. Residual amounts of extraction reagent were removed by dialyzing the antigen solution twice against 1,000 mL 0.9% NaCl and once against 1,000 mL PBS at 4 °C, with an exclusion limit of 12–14 kDa (12–14 kDa dialysis tube, NeoLab, Heidelberg, Germany). The protein concentration of the dialysate was determined using the Advanced Protein Assay Reagent (5x Concentrate, Cycloskeleton Inc., Denver, CO, USA) according to the manufacturer’s instructions.

### Purification of specific anti-*Brachyspira* antibodies from hyperimmune rabbit antisera

Specific antisera against *B. pilosicoli* and *“B. canis”* were raised in rabbits using the antigens extracted as described above (Biogenes commercial antibody production service, Berlin, Germany). A total of 1.5 mL pre-immunization serum and 20 mL antiserum were harvested from each rabbit.

Antigen-specific antibodies against *B. pilosicoli* were isolated by affinity chromatography and adsorption of cross-reacting antibodies. For this, extracted antigens of “*B. canis”* and *B. pilosicoli* were each bound to CNBr-activated Sepharose 4B (VWR, Dresden, Germany) following the manufacturer’s instructions, yielding a “*B. canis*” and a *B. pilosicoli* gel.

For antibody purification, 4.4 mL anti-*B. pilosicoli* antiserum mixed with 500 µL 10x PBS and 100 µL 500 mM EDTA were incubated with 2 mL *B. pilosicoli* gel for 2 h at room temperature (RT). After a washing step with PBS until no protein was detected in the flow-through (OD_280nm_ < 0.05), antibodies bound to the gel were eluted with 0.1 M citrate buffer (pH 2.5) and immediately neutralized (pH 7.5) using 500 mM Na_3_HPO_4_. To eliminate cross-reacting antibodies, 5 mL of the eluate was incubated with 2 mL of “*B. canis”* gel for 1 h at RT. The flow-through with specific antibodies was concentrated by centrifugal filtration at an exclusion limit of 10 kDa (10 kDa centrifugal filter, Vivaspin, VWR).

Isolation of antigen-specific antibodies against “*B. canis”* was performed in a similar fashion, where the first incubation with *“B. canis”* gel was followed by the elimination of cross-reacting antibodies with the *B. pilosicoli* gel.

### *Brachyspira* immunohistochemistry of biopsies from a *Brachyspira*-positive dog

Routine diagnostic endoscopic tissue biopsies were collected from one of the Beagle dogs with chronic signs of gastrointestinal disease. The biopsies were subsequently fixed for 24 h in 4% neutral buffered formaldehyde, automatically processed, embedded in paraffin, cut at 2–4 µM thickness and mounted on glass slides.

Immunohistochemistry was performed on the biopsies from the colon and duodenum as previously described [41, 42]. Briefly, sections mounted on Superfrost^TM^Plus slides (Epredia Netherlands B.V., Breda, Netherlands) were dewaxed, rehydrated, and endogenous peroxidase was blocked with 0.5% H_2_O_2_ (v/v) in methanol for 30 min. Non-specific background signal was blocked with 5% normal goat serum for 30 min. Slides were incubated with the affinity-purified rabbit anti-*B. pilosicoli*-antibody diluted at 1:10,000 or rabbit anti-“*B. canis”*-antibody diluted at 1:1,000 at 4 °C overnight. Subsequently, slides were incubated with secondary biotinylated goat-anti-rabbit-antibodies diluted at 1:200 (BA-1000, Vector Laboratories, Burlingame, CA, USA) at room temperature for 30 min, the avidin-biotin-peroxidase-complex (VEC-PK-6100, Vector Laboratories) for 30 min, and the chromogen diaminobenzidine (DAB). Finally, slides were counterstained with hematoxylin and mounted with Eukitt^®^ quick-hardening mounting medium (ORSAtec GmbH, Bobingen, Germany) and glass coverslips.

Cell pellets of *B. pilosicoli* and “*B. canis”*, as well as archived porcine colonic specimens, previously tested positive for *B. pilosicoli* by PCR, served as positive controls (Fig. 2G-I). For the negative controls, primary antibodies were replaced by normal rabbit serum diluted at 1:100 (011-000-120, Jackson ImmunoResearch Laboratories, West Grove, PA, USA).

Light microscopy was performed with an Olympus BX53 microscope (Evident, Tokyo, Japan) with WHN10X-H/22 oculars, 4x/0.13, 10x/0.30, 20x/0.50, 40x/0.75, and 100x/1.30 oil-immersion objectives and differential-interference-contrast. Digital images were acquired using a 5-megapixel Olympus DP26 color camera and Cell Sens Dimension v. 1.18 software.

### Electronic supplementary material

Below is the link to the electronic supplementary material.

Supplementary Material 1

Supplementary Material 2

## Data Availability

All data analyzed or generated during this study are included in this published article and its supplementary files. Original data (anonymized for patient and owner details) are available from the authors upon reasonable request. *Nox* sequences and *16 S rRNA* sequences not showing 100% identity with any published sequences were submitted to GenBank (see Suppl. Table S2 for accession no.).
