# Supplementary material for: Brachyspira in dogs: risk factors of shedding in central Germany and longitudinal study of an infected kennel
Source: BMC Vet Res. 2024 Apr 4;20:136. doi: 10.1186/s12917-024-03989-x (PMC10993570; doi:10.1186/s12917-024-03989-x)
Supplement: Supplementary file 1 — Supplementary Material 1 [file 12917_2024_3989_MOESM1_ESM.pdf]

UNIVERSITÄT LEIPZIG

Brachyspira in dogs: risk factors of shedding in central Germany

Date of sampling: ______________________________

Practice/clinic where the sample was obtained: ______________________________

Patient

Name: ______________________ Clinic ID: ______________________

Breed: ______________________________

Age: ______________ Sex: ______________ neutered: ☐ yes ☐ no

Body weight: ______________ Body condition score: ______________

Is the dog used for hunting? ☐ yes ☐ no

Is the dog kept in a kennel? ☐ yes ☐ no

Does the dog have access to areas that allow for contact to rodents or other wildlife (e.g., regular walks in the forest, off-leash running in lake areas, etc.)?
☐ yes ☐ no

Is the dog being regularly dewormed? ☐ yes ☐ no
If yes, which medication: ______________________________

Has the dog been regularly vaccinated? ☐ yes ☐ no

If yes, which vaccine: ______________________________

Has the dog ever traveled abroad? ☐ yes ☐ no

If yes, where and when: ______________________________

Owner´s name: ______________________________

UNIVERSITÄT LEIPZIG

Medication history

Were **antimicrobials** administered in the last 3 months before the presentation? ☐ yes ☐ no

If yes, for what indication? ____

____

If yes, please list the antimicrobial(s) used:

Antimicrobial	Dose	Route of administration	First administration (date)	Treatment duration

Were anti-inflammatory drugs administered in the past 3 months prior to presentation?

☐ yes ☐ no If yes, which anti-inflammatory drug: ____________________

____

Were probiotics and/or prebiotics administered in the past 3 months prior to presentation?

☐ yes ☐ no If yes, which pre-/probiotic(s): ____________________

____

Has a fecal sample already been obtained and submitted for fecal diagnostic testing?

☐ yes ☐ no If yes, please attach the results.

Has a clinicopathologic evaluation already been performed or initiated (e.g., hematology, serum biochemistry profile, urinalysis, gastrointestinal panel)?

☐ yes ☐ no If yes, please attach the results.

Medical history and current clinical signs

Defecation frequency

- ☐ 1× daily
- ☐ 2–3× daily
- ☐ 4–5× daily
- ☐ more than 5× daily
- ☐ defecation not observed

Fecal consistency

- ☐ liquid feces
- ☐ soft, unformed feces
- ☐ soft, formed feces
- ☐ firm, well-formed feces (optimal consistency)
- ☐ dry, hard feces
- ☐ defecation not observed

Admixture of blood: ☐ yes ☐ no

Admixture of mucus: ☐ yes ☐ no

Foreign material: ☐ yes ☐ no

Does the dog appear to experience abdominal pain? ☐ yes ☐ no

Does the dog show flatulence (passing gas)? ☐ yes ☐ no

Other gastrointestinal signs

Does the dog have a diagnosed or suspected chronic gastrointestinal condition:

☐ yes ☐ no

Does the dog have vomiting: ☐ yes ☐ no

If yes:
- ☐ 1× per week
- ☐ 2–3× per week
- ☐ more than 3× per week

Does the dog have weight loss: ☐ yes ☐ no

Has fecal testing been previously performed and are the results available?

☐ yes ☐ no

If yes, please attach the results.

Diet history

☐ **commercially available dog food** ☐ **home-made diet** ☐ **complementary food**

A) Commercially available dog food

Dry food: ☐ yes ☐ no

If yes: ☐ portioned ☐ ad libitum

Brand:____________________ ____

Wet/canned food: ☐ yes ☐ no

If yes, brand: ____

B) Home-made diet

☐ portioned ☐ ad libitum ☐ BARF/RMBD

Ingredients: ____

____

C) Dietary supplements:

☐ regularly ☐ occasionally

If yes, please specify: ____

____

Has there been a change in the diet in the last 3 months? ☐ yes ☐ no

If yes, please list the reason(s): ____

____

____

____

Thank you!
