# Supplementary material for: Brachyspira in dogs: risk factors of shedding in central Germany and longitudinal study of an infected kennel
Source: BMC Vet Res. 2024 Apr 4;20:136. doi: 10.1186/s12917-024-03989-x (PMC10993570; doi:10.1186/s12917-024-03989-x)
Supplement: Supplementary file 2 — Supplementary Material 2 [file 12917_2024_3989_MOESM2_ESM.docx]

**Supplementary Material**

**Supplementary Table 1.** Association of *B. canis* and *B. pulli* isolation rates with the dogs’ signalment, demographic variables, medical and diet history, and current health status.

| Variable | *B. canis* isolated (in %) | *B. pulli* isolated (in %) | *Brachyspira* not isolated (in %) | *P*_univariate_  *B. canis* | *P*_multivariate_  *B. canis* | *P*_univariate_  *B. pulli* | *P*_multivariate_ *B. pulli* |
| --- | --- | --- | --- | --- | --- | --- | --- |
| Patient characteristics |  |  |  |  |  |  |  |
| Age <1 year | 7/18 (39%) | 1/6 (17%) | 26/206 (13%) | **0.0080** | **0.0039** | 0.7780 |  |
| Male gender | 6/15 (40%) | 1/6 (17%) | 110/205 (54%) | 0.3060 |  | 0.0638 |  |
| Neutered/spayed | 6/18 (33%) | 4/6 (67%) | 62/200 (31%) | 0.8387 |  | 0.0777 |  |
| Mixed breed | 13/18 (72%) | 4/6 (67%) | 61/201 (30%) | **0.0005** | 0.6533 | 0.0719 |  |
| Body weight  <10 kg  10–25 kg  >25 kg | 1/4 (25%),  2/4 (50%),  1/4 (25%) | 1/3 (33%),  2/3 (67%),  0/3 (0%) | 44/174 (25%), 88/174 (51%), 42/174 (24%) | 0.9992 |  | 0.4402 |  |
| BCS <5 (scale of 9) | 1/2 (50%) | 0 | 15/57 (26%) | 0.4845 |  | - |  |
| Environmental & lifestyle factors |  |  |  |  |  |  |  |
| Shelter origin | 14/18 (78%) | 3/6 (50%) | 43/209 (21%) | **<0.0001** | **<0.0001** | 0.1165 |  |
| Hunting dog | 1/18 (6%) | 1/6 (17%) | 84/206 (41%) | **0.0008** | 0.4441 | 0.2080 |  |
| Kennel dog | 14/18 (78%) | 3/6 (50%) | 77/202 (38%) | **0.0010** | 0.0913 | 0.5608 |  |
| Leashed walks | 9/16 (56%) | 4/6 (67%) | 155/197 (79%) | 0.0554 |  | 0.5038 |  |
| Travel/stay abroad | 5/11 (46%) | 5/6 (83%) | 68/192 (35%) | 0.5061 |  | **0.0173** | **0.0169** |
| Medical and diet history |  |  |  |  |  |  |  |
| Prior antibiotics | 6/16 (38%) | 3/6 (50%) | 60/198 (30%) | 0.5552 |  | 0.3223 |  |
| Pre-or probiotics | 1/13 (8%) | 0/5 (0%) | 18/171 (11%) | 0.7365 |  | 0.2953 |  |
| Vaccination | 16/18 (89%) | 6/6 (100%) | 186/204 (91%) | 0.7524 |  | 0.2961 |  |
| Deworming | 17/18 (94%) | 6/6 (100%) | 168/200 (84%) | 0.1863 |  | 0.1513 |  |
| Anti-inflammatories  -NSAID  -Steroid | 2/12 (17%)  1/12 (8%)  1/12 (8%) | 2/4 (50%)  1/4 (25%)  1/4 (25%) | 45/175 (26%)  27/175 (15%)  8/175 (5%) | 0.4662  0.4749  0.5893 |  | 0.3058  0.6254  0.1715 |  |
| Raw feeding (BARF diet) | 1/14 (7%) | 0/6 (0%) | 10/175 (6%) | 0.8312 |  | 0.4048 |  |
| Recent diet change | 2/8 (25%) | 2/4 (25%) | 43/156 (28%) | 0.8729 |  | 0.3498 |  |
| Kibble diet | 13/13 (100%) | 5/6 (83%) | 150/167 (90%) | 0.1015 |  | 0.6338 |  |
| Wet/canned diet | 6/13 (46%) | 3/6 (50%) | 79/167 (47%) | 0.9361 |  | 0.8967 |  |
| Commercial diet | 14/15 (93%) | 6/6 (100%) | 174/184 (95%) | 0.8452 |  | 0.4167 |  |
| Home-made diet | 1/15 (7%) | 0/6 (0%) | 44/184 (24%) | 0.0844 |  | 0.0728 |  |
| Dietary supplements | 3/15 (20%) | 1/6 (17%) | 25/184 (14%) | 0.5121 |  | 0.8335 |  |
| Co-infection w/ Giardia and/or Cryptosporidium | 0/2 (0%) | 0/2 (0%) | 7/19 (37%) | 0.1890 |  | 0.1658 |  |
| Clinical signs of gastrointestinal disease |  |  |  |  |  |  |  |
| Known or suspected chronic enteropathy | 2/18 (11%) | 2/6 (33%) | 33/187 (18%) | 0.4597 |  | 0.3646 |  |
| Defecation frequency* | 1 (0–2) | 1 (1–2) | 1 (0–3) | 0.5648 |  | 0.6453 |  |
| Fecal consistency AHDi*  Waltham score | 0.25 (0–2.5)  3.75 (1.5–4) | 0.25 (0–0.5)  3.75 (3.5–4) | 0 (0–3)  4 (1–4.5) | 0.8887  0.8727 |  | 0.5914  0.6010 |  |
| Hematochezia/melena  Mucus  undigested material | 0/18 (0%)  0/18 (0%)  0/18 (0%) | 0/6 (0%)  1/6 (17%)  0/6 (0%) | 11/190 (6%)  19/188 (10%)  9/185 (5%) | 0.1524  0.0559  0.1908 |  | 0.4014  0.6286  0.4429 |  |
| Flatulence | 0/6 (0%) | 0/5 (0%) | 32/163 (20%) | 0.1089 |  | 0.1427 |  |
| Abdominal pain | 1/7 (14%) | 1/5 (20%) | 13/166 (8%) | 0.5947 |  | 0.3985 |  |
| Weight loss | 2/17 (12%) | 0/6 (0%) | 16/183 (9%) | 0.6884 |  | 0.2988 |  |
| Vomiting | 0/18 (0%) | 2/6 (33%) | 15/172 (9%) | 0.0775 |  | 0.1004 |  |
| Vomiting score* | 0 (0) | 0 (0-1) | 0 (0–1) | 0.1932 |  | **0.0462** | 0.0894 |
| Clinical severity score* | 1.25 (0–4.5) | 1.5 (1–3.5) | 1.5 (0–7) | 0.8749 |  | 0.6136 |  |
| At least one GI sign^‡^ | 9/17 (53%) | 4/6 (67%) | 105/195 (54%) | 0.9428 |  | 0.5297 |  |

*criterion or criteria of the acute hemorrhagic diarrhea index (AHDi; [24]); ‡includes diarrhea (and/or hematochezia/melena, mucus, other material), vomiting, weight loss, abdominal pain, and/or flatulence.

**Supplementary Table 2**. Overview of the sequenced genes (*nox* and *16S rRNA*) with less than 100% identity after BLAST analysis, which were admitted to the GenBank via BankIt (<https://submit.ncbi.nlm.nih.gov/about/bankit/>) and subsequently received the listed GenBank accession numbers.

| Isolate no. | Gen | GenBank accession no. |
| --- | --- | --- |
| 88 | *16S rRNA* | OQ625946 |
| 96 | *16S rRNA* | OQ625947 |
| 108 | *16S rRNA* | OQ625948 |
| 178 | *16S rRNA* | OQ625949 |
| 184 | *16S rRNA* | OQ625950 |
| 346 | *16S rRNA* | OQ625951 |
| 88 | *nox* | OQ680200 |
| 102 | *nox* | OQ680206 |
| 108 | *nox* | OQ680201 |
| 177 | *nox* | OQ680207 |
| 178 | *nox* | OQ680202 |
| 184 | *nox* | OQ680203 |
| 303 | *nox* | OQ680204 |
| 346 | *nox* | OQ680205 |

**Supplementary Fig. 1.** Uncropped image** of the agarose gel shown in Fig. 3 generated with the Vilber Lourmat Quantum ST5 1100/26MX and the Software VisionCapt (using the following settings: DNA gels and autoexposure).


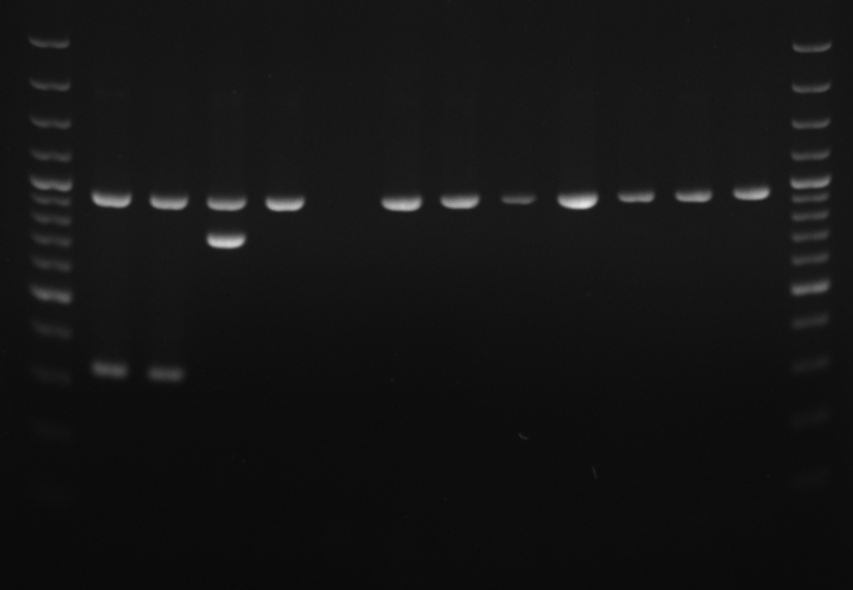


**Please note that the gel documentation system used to generate the picture shown in Fig. 3 generates pictures that do not allow to recognize the borders of the gel. As can be assessed by the marker bands shown in Fig. 3 and Supp. Fig. 1 we did not cut off any parts of the picture to hide any additional bands.
